# VSTH: a user-friendly web server for structure-based virtual screening on Tianhe-2

**DOI:** 10.1093/bioinformatics/btac740

**Published:** 2022-11-17

**Authors:** Qing Mo, Zexin Xu, Hui Yan, Pin Chen, Yutong Lu

**Affiliations:** National Supercomputer Center in Guangzhou, School of Computer Science and Engineering, Sun Yat-Sen University, Guangzhou 510006, China; National Supercomputer Center in Guangzhou, School of Computer Science and Engineering, Sun Yat-Sen University, Guangzhou 510006, China; National Supercomputer Center in Guangzhou, School of Computer Science and Engineering, Sun Yat-Sen University, Guangzhou 510006, China; National Supercomputer Center in Guangzhou, School of Computer Science and Engineering, Sun Yat-Sen University, Guangzhou 510006, China; National Supercomputer Center in Guangzhou, School of Computer Science and Engineering, Sun Yat-Sen University, Guangzhou 510006, China

## Abstract

**Summary:**

VSTH is a user-friendly web server with the complete workflow for virtual screening. By self-customized visualization software, users can interactively prepare protein files, set docking sites as well as view binding conformers in a target protein in a few clicks. We provide serval purchasable ligand libraries for selection. And, we integrate six open-source docking programs as computing engine, or as conformational sampling tools for DLIGAND2. Users can select various docking methods simultaneously and personalize computing parameters. After docking processing, user can filter docking conformations by ranked scores, or cluster-based molecular similarity to find highly populated clusters of low-energy conformations.

**Availability and implementation:**

The VSTH web server is free and open to all users at https://matgen.nscc-gz.cn/VirtualScreening.html

**Supplementary information:**

Supplementary data are available at Bioinformatics online.

## 1. Introduction

Virtual screening (VS) aims at identifying compounds potentially regulating a biological target of interest and, has become a significant component in the pharmaceutical industry for drug discovery. Structure-based molecular docking is one of the computing methods, based on searching molecules with high binding affinity to that of the known binding site of proteins. Recently, web tools have been reported for online molecular docking or screening services. The coronavirus disease 2019 (COVID-19) Docking Server (Kong *et al.*, 2020) focuses on the proteins involved in the COVID-19 virus life cycle, providing an interactive tool for the prediction of COVID-19 target–ligand interactions. The Webina (Kochnev *et al.*, 2020) is a web version of AutoDock Vina (Trott and Olson, 2009) runs docking entirely with a local web browser. EasyVS (Pires *et al.*, 2020) simplifies molecule library selection and structure-based VS. CB-Dock (Liu *et al.*, 2020) can predict binding sites automatically. But these servers cannot personalize to process proteins (e.g. removing specified binding ligands, ions, waters, etc.). In addition, these servers lack the diversity of computing engines.

To address the above limitations, we propose VSTH, a web server that integrates six docking programs, AutoDock Vina, AutoDock4 (Morris *et al.*, 2009), GalaxyDock3 (Shin and Seok, 2012), idock (Li *et al.*, 2012), iGEMDOCK (Yang and Chen, 2004) and ledock (Zhao and Caflisch, 2013). VSTH provides an optional in-house scoring function of DLIGAND2 (Chen *et al.*, 2019), to re-assess the conformations generated by docking software. By self-customized 3D visualization software, users can interactively prepare protein files, set docking sites as well as view binding conformers in a target protein in a few clicks. After docking processing, user can filter docking conformations by ranked score, or cluster-based molecular similarity to find highly populated clusters of low-energy conformations.

## 2. Platform description

**Fig. 1.**
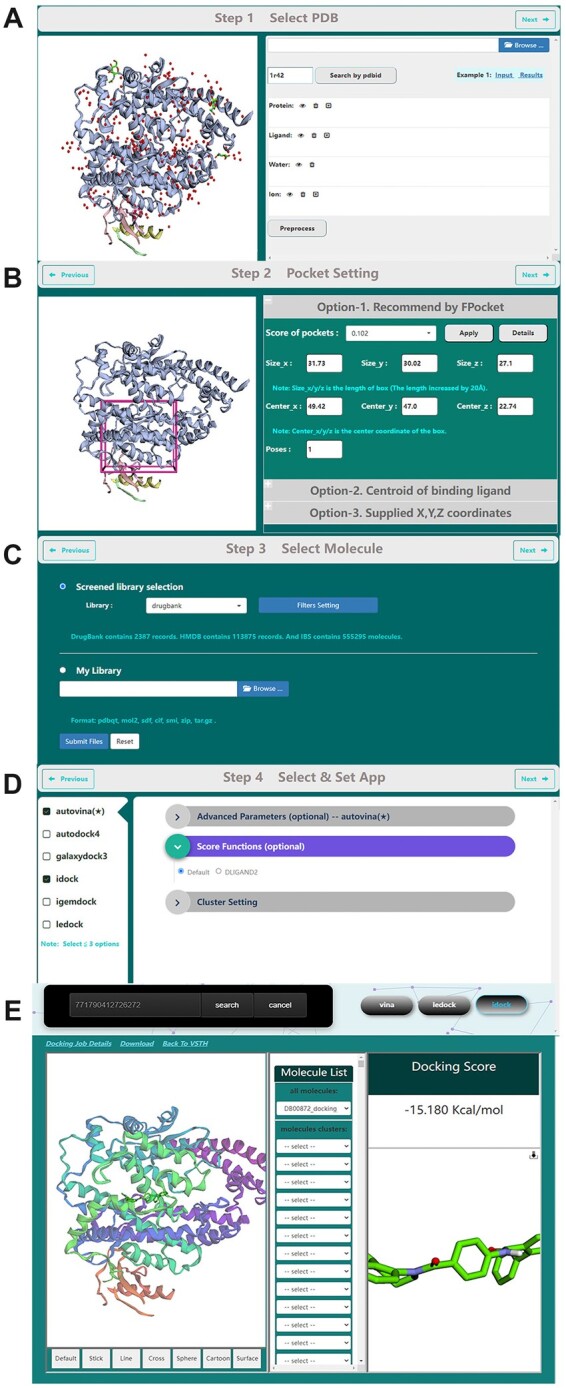
VSTH web page. (**A**) Prepare protein structure. (**B**) Select pocket. (**C**) Select ligand library. (**D**) Select docking programs and set computing parameters. (**E**) Check job status and get job result by task id

The customized 3Dmol.js (Rego and Koes, 2015) is used for 3D proteins visualization and interactively keeping or removing specific protein chains, molecules, waters and ions (Fig. 1A). Reduce (Word *et al.*, 1999) is used for adding hydrogens, rotating and flipping NQH groups. For pocket (Fig. 1B), users can select a pocket from the at most 10 ranked pockets recommended by Fpocket (Le Guilloux *et al.*, 2009), or from the centroid list of the binding ligand, or supply the (X, Y, Z) coordinates of its center and its box size directly. Available public libraries include DrugBank 5.0 (Wishart *et al.*, 2018), HMDB 4.0 (Wishart *et al.*, 2007), InterBioScreen 2020, and there are filters for refining these molecular libraries (Fig. 1C). Users can upload molecule library of interest. Six docking methods are provided, and users have the option to use DLIGAND2 for rescoring (Fig. 1D). VSTH provides a dashboard to check job status, cancel job and get job result (Fig. 1E). Users can also download complex of protein and molecule for downstream tasks (for instance, molecular dynamics). A detailed implementation can be found in Supplementary Information with a flowchart of VSTH (Supplementary Fig. S1) and relevant toolkits we used (Supplementary Table S1).

We also show a step-by-step guide for users in Supplementary Information (Supplementary Figs. S2–S9) and the advanced parameters for six docking programs (Supplementary Table S2). To demonstrate the novelty of VSTH, we compare VSTH with state-of-art structure-based web servers in Supplementary Table S3. Then, we validate the capacities of VSTH in identifying decoys on known binding site and drug repositioning for screening ACE2 enzymatic activators on unknown binding site, which are shown in Supplementary Information. The results indicate (Supplementary Table S4–S6) that rescoring by our in-house DLIGAND2 can improve the ability of identifying the correct poses and ranking power for targeting drugs.

## 3. Results

We present a user-friendly web server with the complete workflow for virtual pharmaceutical screening. VSTH allows users to prepare proteins files, set docking sites, select well-constructed purchasable ligand libraries, use various docking methods simultaneously, personalize computing parameters and perform post-processing analysis. The VSTH server will continue to be developed, integrate more docking software and scoring functions, and we believe it will be a useful tool for VS.

## Supplementary Material

btac740_Supplementary_Data

## Data Availability

Data and software underlying this article are availabe at https://matgen.nscc-gz.cn/wiki/index.php/Virtual_Screening.
