# Supplementary material for: VSTH: a user-friendly web server for structure-based virtual screening on Tianhe-2
Source: Bioinformatics. 2022 Nov 17;39(1):btac740. doi: 10.1093/bioinformatics/btac740 (PMC9805582; doi:10.1093/bioinformatics/btac740)
Supplement: btac740_Supplementary_Data [file btac740_supplementary_data.pdf]

Supplementary Information

VSTH: a user-friendly web server for structure-based virtual screening on Tianhe-2

Qing Mo[1], Zexin Xu[1], Hui Yan[1], Pin Chen*,[1], and Yutong Lu*,[1]

[1] National Supercomputer Center in Guangzhou, School of Computer Science and Engineering, Sun Yat-Sen University, Guangzhou 510006, China

*To whom correspondence should be addressed.

Web Server Implementation

Framework

VSTH can provide a complete workflow for molecular docking. We elaborately design four step-by-step guides to start a docking task through VSTH, users only need to provide a protein structure, molecular library and select docking parameters by clicking mouse on the web page. We display the whole workflow in Figure S1.

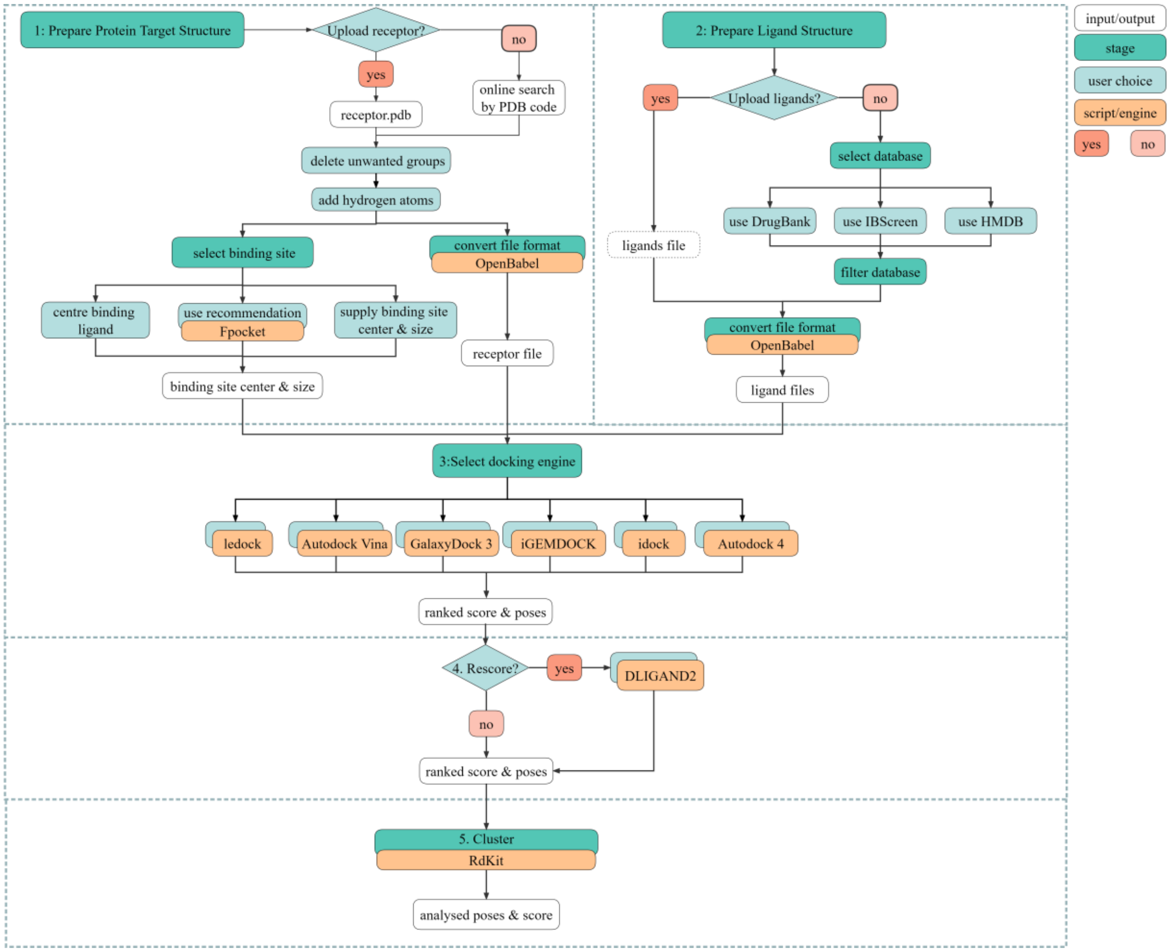

Fig. S1. The workflow of VSTH.

Web Interface

The web interface is written in HTML, JavaScript, and Bootstrap, enabling users to access it from a diversity of browsers, like Chrome 103.0.5060.66, Firefox 101.0.1, Microsoft Edge 103.0.1264.37, Opera 91.0.4516.65 and Safari 15.6.1, which have been tested. 3Dmol.js (Rego and Koes, 2015) is used for 3D proteins visualization and interactively keeping or removing specific side chains, molecules, waters and ions during the protein target preparation step. Then in binding site setting step, it is used to present an interactive viewer of the protein target and the best scoring pocket recommended by Fpocket. In task monitoring page, 3Dmol.js is used to show the protein target and conformations.

Backend

VSTH runs on a Linux server with the 2.2 GHz Intel Xeon processors (28 cores) and 64 GB memory. After users clicked the task submission button, VSTH will queue the task in Redis and return the task identifier on the web page. Our back-end executor will extract the top ranked task in Redis, fork the task into small docking jobs and run these jobs in parallel. Each job results will be analyzed, and the binding affinity scores and corresponding conformations will be pushed into MongoDB for displaying. We provide email reminder for users to inform job status.

The docking results will be clustered based on molecular similarity algorithm implemented in RDKit package when job is finished. On the one hand, it can cluster the same compound with multiple conformations into one class when users set pose argument greater than 1. On the other hand, a subset of similar compounds can be filtered by clustering to further improve the diversity of potential compounds.

Users can download finished job details from VSTH, like conformation score, conformation mol2 file, protein file, and clustering information.

The details of toolkits implemented in VSTH backend are described in Table S1.

Table S1. The toolkits used for VSTH backend development.

Tools	Version	Purpose	Linkage
**MongoDB**	3.2.22	Storage database	https://www.mongodb.com
**Redis**	4.1.6	Memory cache	https://redis.io
**Spring boot**	2.1.6	Web server framework	https:/spring.io
**ActiveMQ**	5.16.0	Messaging server	http://activemq.apache.org
**RDKit**	2020.09.1	Conformation clustering	https://github.com/rdkit/rdkit
**MGL-tools**	1.5.7	Receptor and ligand processing	http://mgltools.scripps.edu
**Reduce**	3.23.13	Hydrogen adding	http://kinemage.biochem.duke.edu/software/reduce

**Open Babel**	2.4.1	File format conversion	http://openbabel.org/wiki/Main_Page
**Fpocket**	3	Binding site detection	https://github.com/Discngine/fpocket
**DLIGAND2**	latest	Rescoring	https://github.com/sysu-yanglab/DLIGAND2
**AutoDock Vina**	1.1.2/1.2.3	Molecular docking	http://vina.scripps.edu
**AutoDock4**	2.5.1	Molecular docking	http://autodock.scripps.edu
**GalaxyDock3**	3(latest)	Molecular docking	http://galaxy.seoklab.org
**LeDock**	latest	Molecular docking	http://www.lephar.com/download.htm
**iGEMDOCK**	1	Molecular docking	http://gemdock.life.nctu.edu.tw/dock/download.php
**idock**	2.2.2	Molecular docking	https://github.com/HongjianLi/idock

Result

VSTH can provide a complete workflow for molecular docking, including PDB file preparation, pocket setting, molecular database preparation, docking program selection, task monitoring, and result analysis and visualization. During the input file preparation process, the end users can follow the prompts on the web page to provide a structure of the protein target by either uploading a PDB file or obtaining online by inputting a PDB code. Users also can delete water, ion, ligand and specific side chains as they want (Fig. S2).

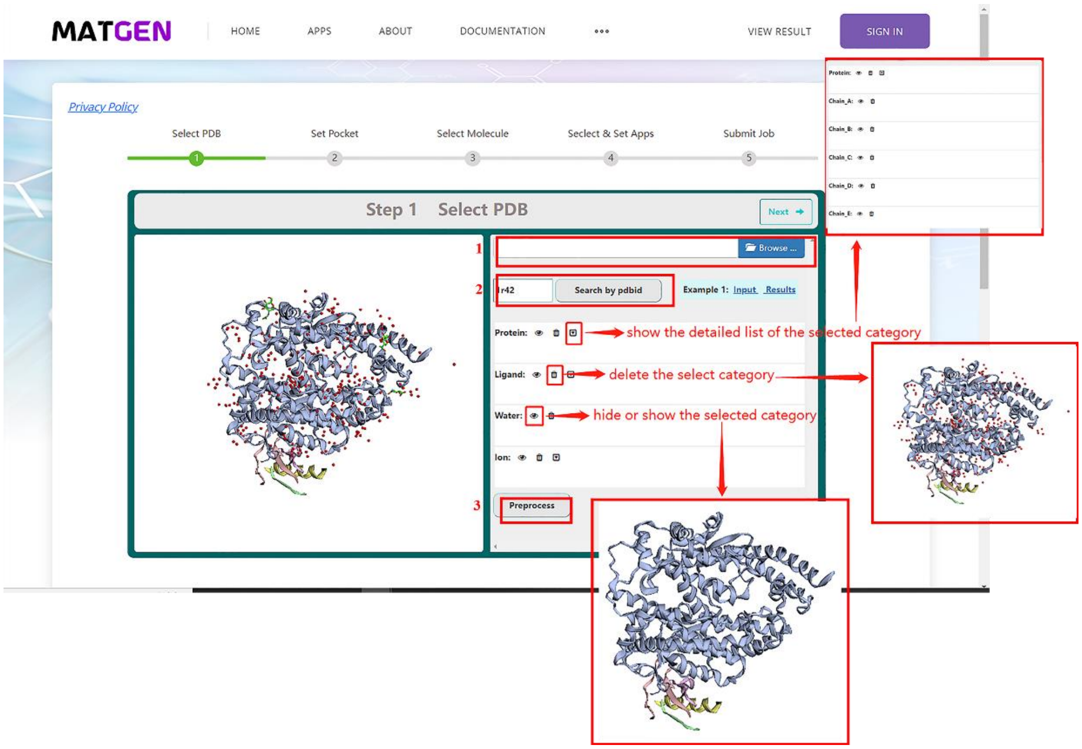

Fig. S2. The snapshot of selecting protein.

What's more, users can add hydrogen to the target protein by clicking the "Preprocess" button. There are 3 types of adding hydrogen, add H and rotate and flip NQH groups; add H and rotate groups with no NQH flips and add H, including His sc NH, then rotate and flip groups Fig. S3).

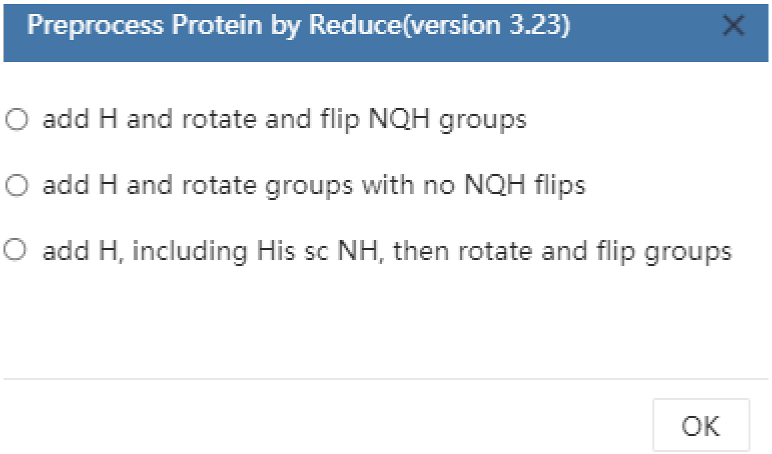

Fig. S3. The snapshot of preprocessing.

VSTH provides three methods for defining targets: Option-1, a list of binding sites recommended by Fpocket(Le Guilloux, et al., 2009) and automatically present an interactive viewer of the protein target and the best scoring pocket. Users can select binding site from the at most 10 ranked pockets. Option-2, users can define the binding site by the centroid of the binding ligand. Option-3, users can supply the (X, Y, Z) coordinates of its center and its box size directly. What's more, users can define the number of poses. (Fig. S4).

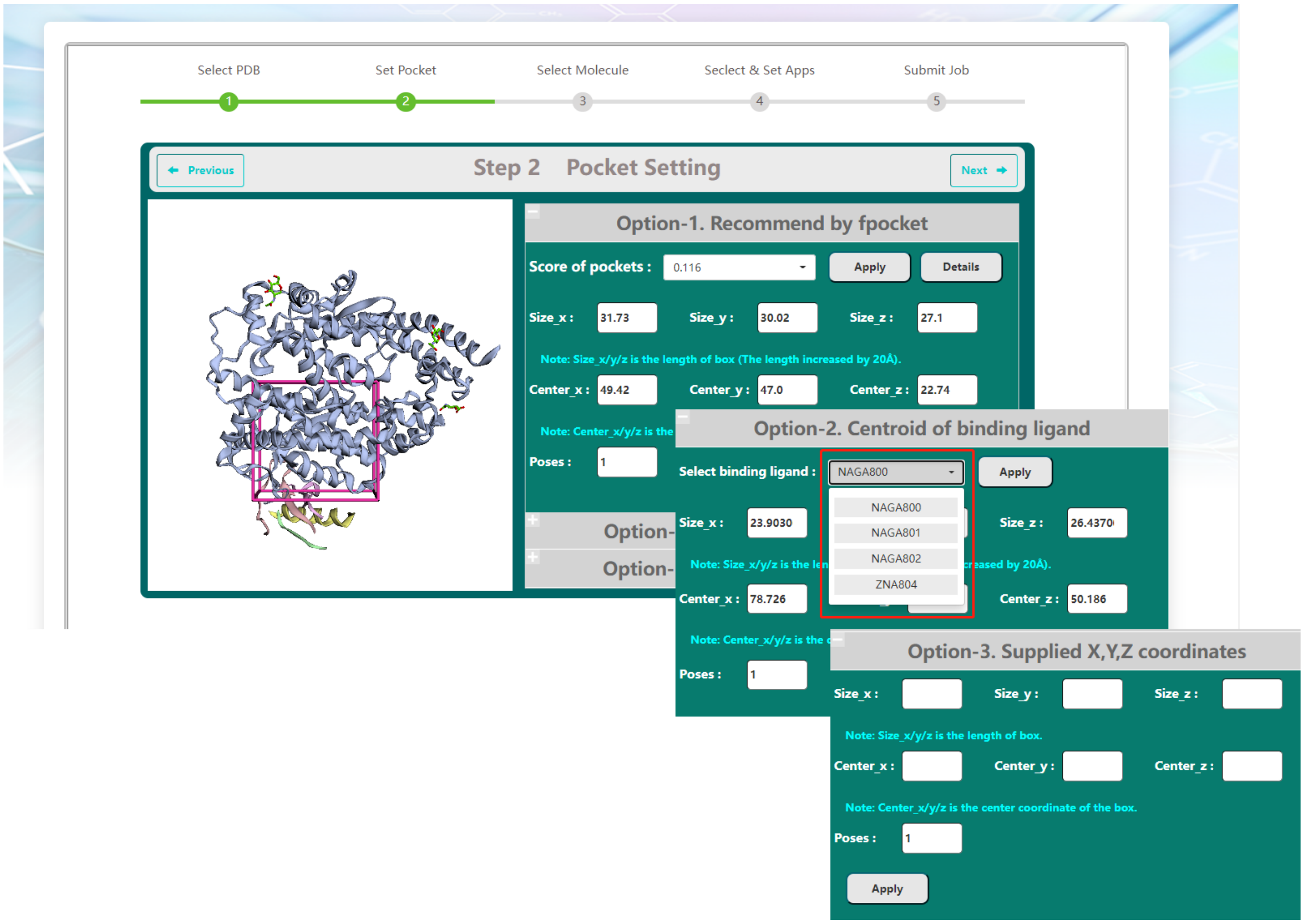

Fig. S4. The snapshot of setting pocket setting.

Step 3 allows users to select libraries. Available public libraries in VSTH include DrugBank5.0 (approved drug dataset, comprises 2387 molecules)(Wishart et al., 2018), HMDB4.0 (comprises 13,875 molecules)(Wishart et al., 2007), and InterBioScreen 2020 (purchased nature compound dataset and synthetic compounds dataset vended by InterBioScreen Company, comprises 555,295 molecules). There are many filters available for refining these molecular libraries, including by topological polar surface area, molecular weight, logP, number of acceptors or donors of hydrogen, rotatable bonds and rings. Users can also upload their own ligand libraries. VSTH supports file formats like pdbqt, mol2, mol, sdf, sd, and smi. If more files are used, users can compress these files in zip or tar.gz format. (Fig. S5 and Fig. S6).

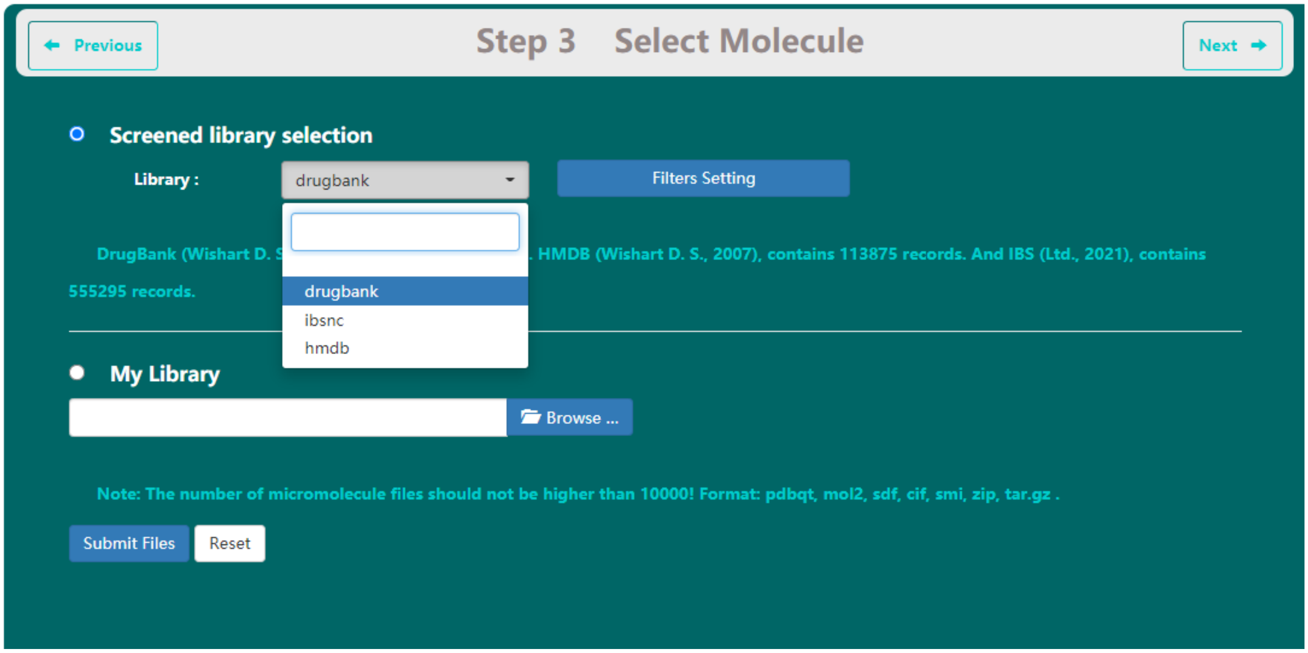

Fig. S5. The snapshot of ligand library selection.

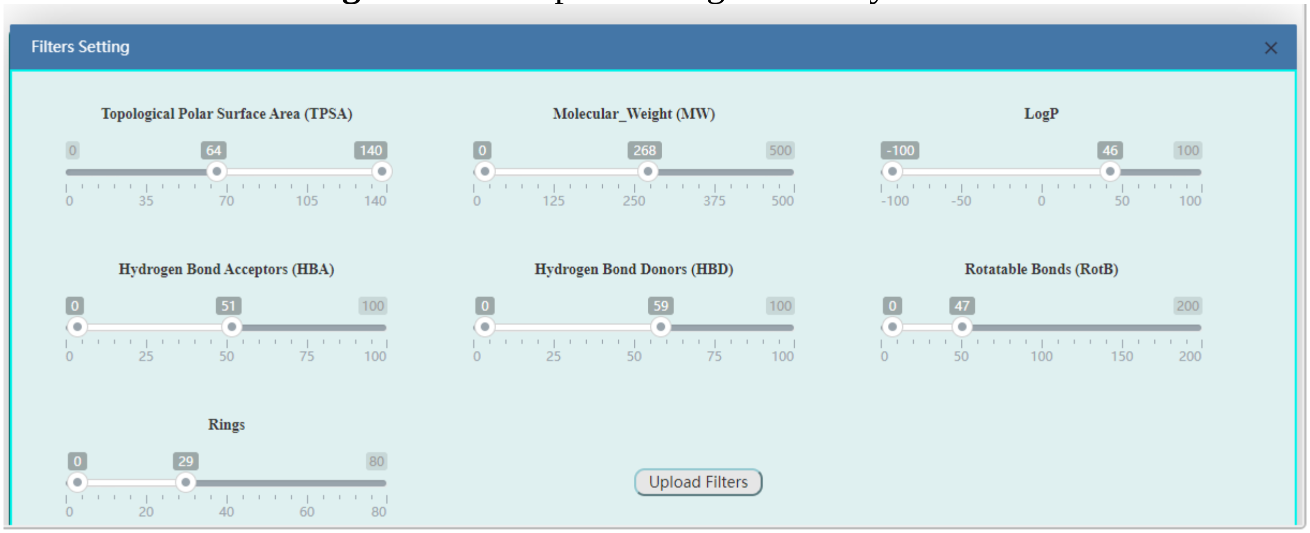

Fig. S6. The snapshot of filtering ligands.

Step 4 allows users to select docking programs and set parameters. VSTH provides 6 docking programs (1). We provide two versions for Autodock Vina. The v1.1.2(Trott and Olson, 2009) is the default, and the latest version v1.2.3(Eberhardt, et al., 2021) can be chosen by users. Users can select at most 3 programs at one time. Generally, VSTH provides the default parameters for docking, while users can modify parameters for personalized docking (2). All advanced parameters are provided according to docking programs and can be found in Table 2. Besides these advanced parameters, users have the option to select DLIGAND2(Chen et al., 2019) to re-score the docking conformations. For conformation classification, users can set the cutoff argument (Fig. S7).

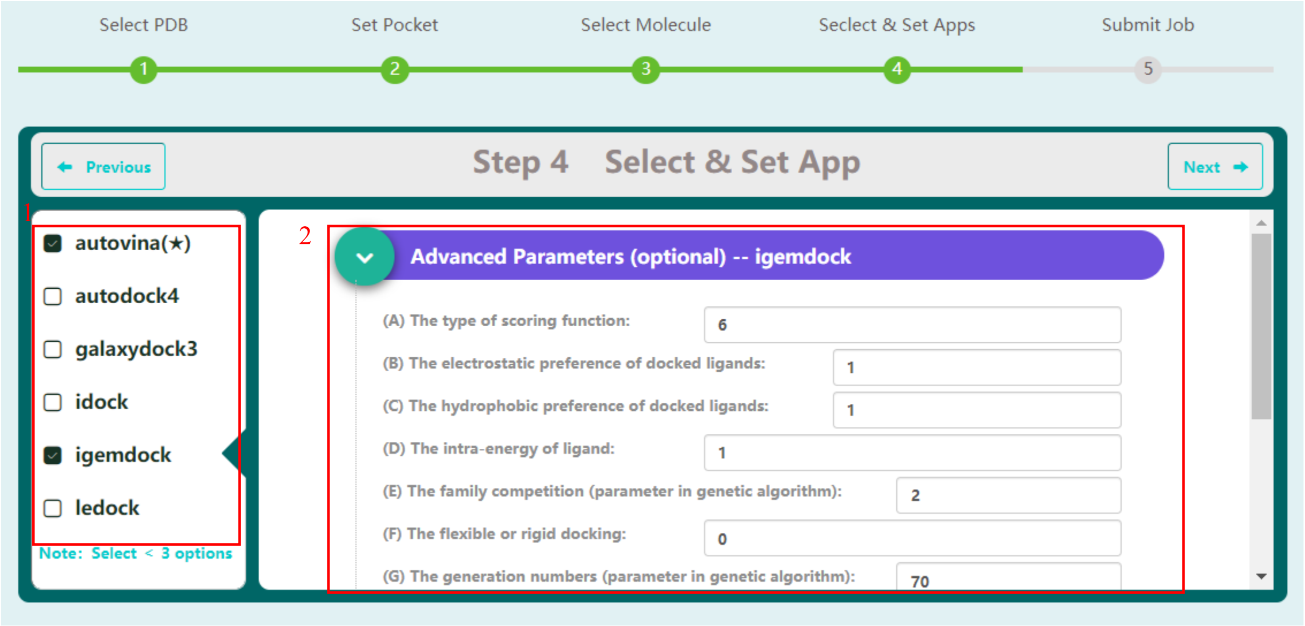

Fig. S7. The snapshot of selecting docking program and setting parameters.

Table S2. Six docking programs and their advanced parameters.

Docking programs	Advanced parameter	Default value
**AutoDock Vina**	The exhaustiveness arg.	8
	The energy range arg.	3
	The seed arg.	1612330277
	The weight_gauss1 arg.	-0.035579
	The weight_gauss2 arg.	-0.005156
	The weight_repulsion arg.	0.840245
	The weight_hydrophobic arg.	-0.035069
	The weight_hydrogen arg.	-0.587439
	The weight_rot arg.	0.05846
	The min_rmsd arg.(for v1.2.3)	1
	The weight_glue arg. (for v1.2.3)	50
	The weight_vinardo_gauss2 arg. (for v1.2.3)	-0.005156
	The weight_vinardo_repulsion arg. (for v1.2.3)	0.840245
	The weight_vinardo_hydrophobic arg. (for v1.2.3)	-0.035069
	The weight_vinardo_hydrogen arg. (for v1.2.3)	-0.587439
	The weight_vinardo_rot arg. (for v1.2.3)	0.05846
**AutoDock4**	----	----
**GalaxyDock3**	The e1 max E_CUTOFF	1000000

	The e0 max E_CUTOFF	1000
**idock**	The seed arg.	1612330277
	The trees arg.	500
	The tasks arg.	64
	The granularity arg.	0.125
**iGEMDOCK**	The type of scoring function	6
	The electrostatic preference of docked ligands	1
	Hydrophobic preference of docked ligands	1
	Intra-energy of ligand	1
	Family competition (parameter in genetic algorithm)	2
	Flexible or rigid docking	0
	The population size	300
**ledock**	----	----

On Step 5, users have the option to type in their email. This email address will be used to receive the task id and get reminder when the task has been finished or cancelled (Fig. S8 and Fig. S9).

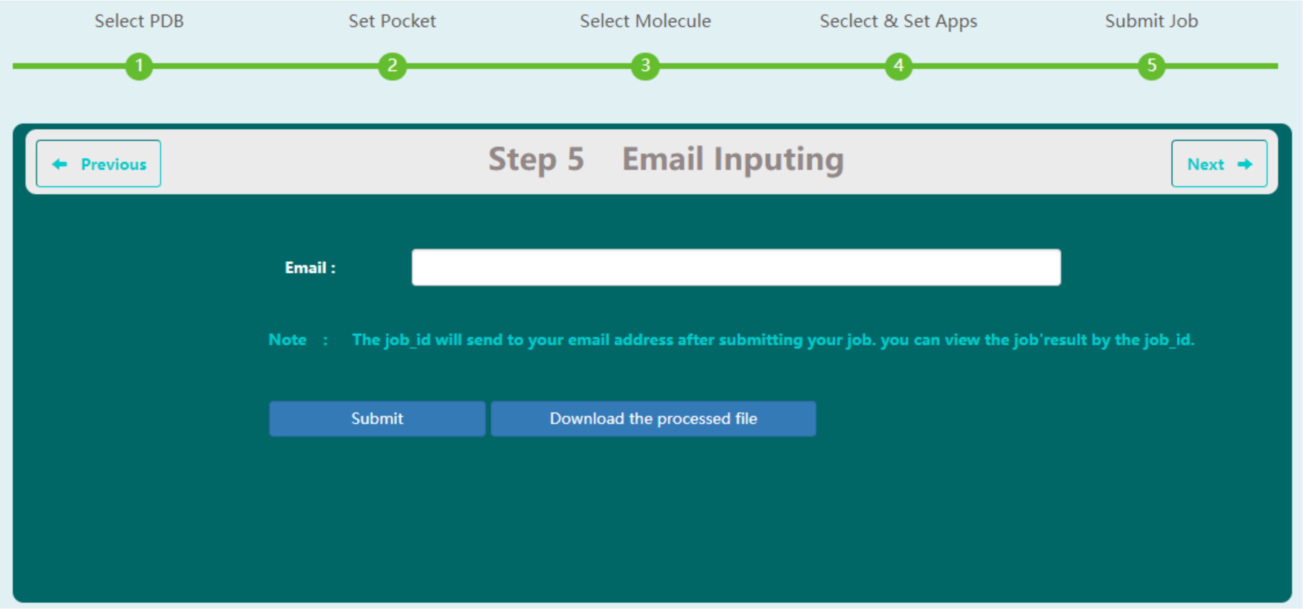

Fig. S8. The snapshot of email inputing.

We design a dashboard to check job status, and users need to provide a task identifier to obtain the specified job. Based on process of the job on the back-end server, we provide status of pending, running and finish. Users can also cancel the job on the dashboard due to some errors. When the job is finished, users can download the protein file, conformation file and the related docking score.

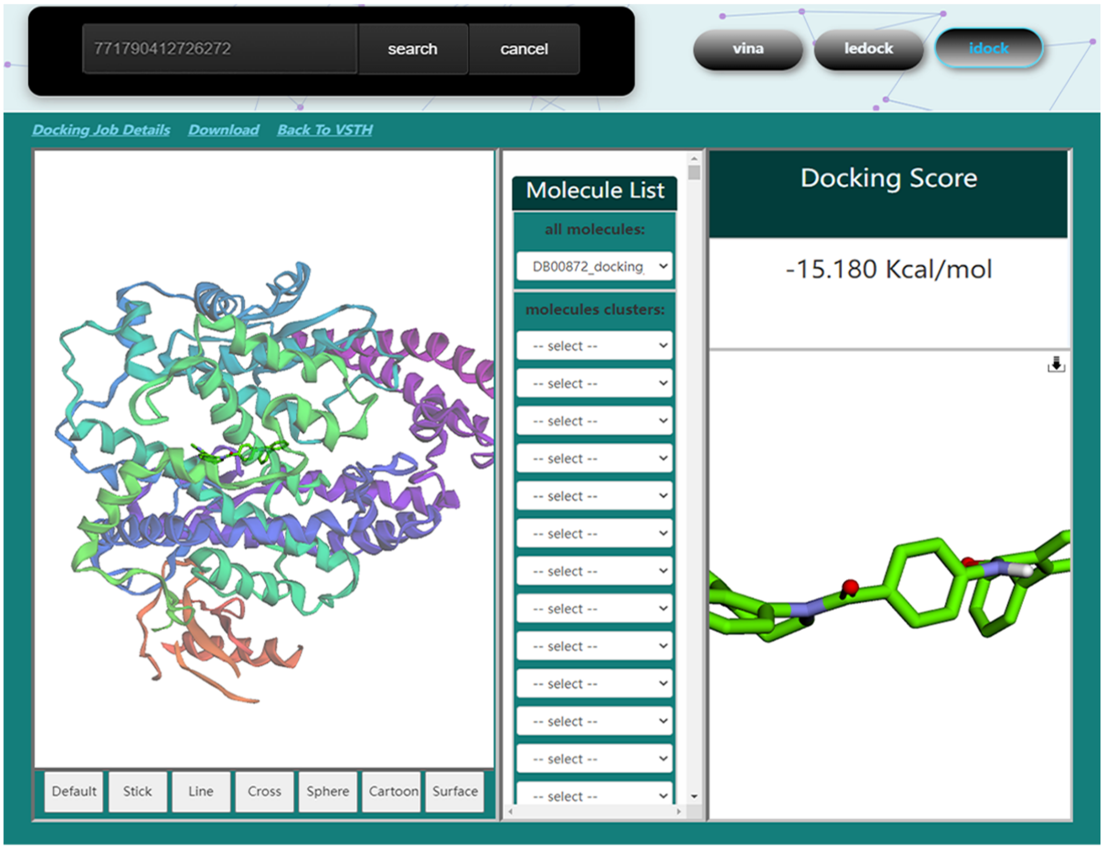

Fig. S9. The snapshot of task monitoring.

Comparisons with other web-based VS programs

As shown in Table S3, we compare VSTH with state-of-art structure-based web servers based on functions for the whole VS process. One unique function of VSTH is to allow online processing of proteins, which ensures the integrity of the data flow during the VS process and does not need to process any data locally. Another feature of VSTH is that it provides multiple docking engines and a scoring function for re-scoring, which improves the docking prediction reliability of VSTH. By synthesizing the results of multiple docking engines and rescoring the docking poses, it is possible to avoid the inclination of docking software to a certain type of proteins in the design process.

Table S3. Comparisons with state-of-art molecular docking web servers.

Server	Description					Published Time
	Interactively processing protein	Providing Ligand Library	Docking Engine	Rescoring	Compounds Clustering	
VSTH	√	√	√ (6)	√	√	2022
EasyVS	X	√	√ (1)	X	√	2021
Webina	X	X	√ (1)	X	X	2020
COVID-19 Docking Server	X	X	√ (1)	√	X	2020

CB-Dock	X	X	√ (1)	X	X	2019

Case Study

We will show the capacities of VSTH in identifying decoys on known binding site and screening potential compounds on unknown binding site in the following evaluations

Study 1: Evaluating VSTH on targets with known binding site

To assess the performance of docking methods in predicting the structures of protein complexes, we used two benchmarks. We first docked 10 receptor-ligand complexes obtained from DUD-E database(Mysinger et al., 2012) using AutoDock Vina and re-assessed these docking poses by DLIGAND2. The average root-mean-square deviations (RMSD) for AutoDock Vina and DLIGAND2 are 5.47 Å and 2.1Å (Supplementary Table S4), indicating that DLIGAND2 can improve the ability of identifying the correct poses. We then used decoy libraries for the same data set to validate the ability of separating true molecules from decoys. DLIGAND2 achieved the better enrichment factor (EF) than AutoDock Vina with $EF_{1\%}$ of 10.01, $EF_{5\%}$ of 4.31 and $EF_{10\%}$ of 2.95, respectively, which is shown in Table S5.

Table S4. Comparison of docking poses between AutoDock Vina (Vina) and DLIGAND2.

Target name	PDB	Ligand	Method	Score (Kcal/mol)	RMSD
ADA	2e1w	FR6	Vina	-9.3	6.44
			DLIGAND2	-7.9	1.47
AKT1	3cqw	CQW	Vina	-8.5	0.40
			DLIGAND2	-8.3	0.40
CP2C9	1r9o	FLP	Vina	-9.1	2.18
			DLIGAND2	-6.6	0.82
FKB1A	1j4h	SUB	Vina	-7.5	7.21
			DLIGAND2	-11.6	1.27
MP2K1	3eqh	5BM	Vina	-9	8.47
			DLIGAND2	-10.9	3.13
HIVINT	3nf7	CIW	Vina	-7.6	8.26
			DLIGAND2	-6.4	4.49
HIVPR	1xl2	189	Vina	-9.7	6.91
			DLIGAND2	-10.1	5.85
HS90A	1uyg	PU2	Vina	-8.4	5.46
			DLIGAND2	-9.4	0.80
RENI	3g6z	A7T	Vina	-10.9	0.69
			DLIGAND2	-17.5	0.69
TRY1	2ayw	ONO	Vina	-8.4	8.69
			DLIGAND2	-11.8	2.08

Table S5. Comparison of $EF_{x\%}$（x=1, 5, 10）between DLIGAND2 and AutoDock Vina (Vina).

Target name	PDB	Ligand	$EF_{1\%}$ DLIGAND2	$EF_{1\%}$ Vina	$EF_{5\%}$ DLIGAND2	$EF_{5\%}$ Vina	$EF_{10\%}$ DLIGAND2	$EF_{10\%}$ Vina
ADA	2e1w	FR6	4.62	1.94	1.37	0.88	0.99	0.93
AKT1	3cqw	CQW	17.53	2.53	7.41	2.41	4.36	1.93
CP2C9	1r9o	FLP	9.88	0.98	2.94	2.14	2.01	2.01
FKB1A	1j4h	SUB	9.54	6.62	4.02	3.53	2.2	2.20
HIVINT	3nf7	CIW	14.74	2.25	6.34	1.38	4.53	1.59
HIVPR	1xl2	189	16.94	2.17	6.84	1.87	4.59	1.80
HS90A	1uyg	PU2	0	0	0.16	0	0.159	0.14
MP2K1	3eqh	5BM	7.03	1.36	3.79	0.91	3.42	0.96
RENI	3g6z	A7T	12.26	3.94	5.62	1.67	3.95	1.25
TRY1	2ayw	ONO	7.52	2.84	4.56	2.73	3.31	2.87

We chose three regions of EF in top x% of the dataset, where x equals to 1, 5 and 10 respectively.

$$EF_{x\%} = \frac{N_{True}^{x\%} / N_{Selected}^{x\%}}{N_{Active} / N_{Total}} \quad (1)$$

Where $N_{True}^{x\%}$, $N_{Selected}^{x\%}$, N_{Active} and N_{Total} are the number of true positives, the number of selected candidates at top x% screened candidates, the number of active compounds, and the total number of compounds in the screened library, respectively.

Study 2: Evaluating VSTH on targets with unknown binding site

Previous study reported that approved drugs (imatinib, methazolamide) have been repositioning as the ACE2 enzymatic activators to ameliorate COVID-19-induced metabolic complications(Li, et al., 2022). Here, we validate the server using ACE2 (PDB code: 1R42) by selecting the binding site located in the hinge-bending region of protein to screen DrugBank database.

As shown in Table S6, we tested all docking engines and re-scored all conformations by DLIGAND2. We found that GalaxyDock(Shin and Seok, 2012) is the fastest to screen all approved drugs in Drugbank, while iGEMDOCK(Yang and Chen, 2004) is quite slow. We used the default population size of 300 in iGEMDOCK, and reducing this value can speed up the calculation. The affinities predicted by the 6 docking engines are quite different, LeDock(Zhao and Caflisch, 2013) and idock(Li, et al., 2012) show the best ranking power to identify methazolamide and imatinib, respectively. In addition, DLIGAND2 improve ranking power for all docking engines except idock for imatinib.

Table S6. Evaluation on targets with unknown binding ligand

Docking engine	Score Method	Cost time (core hours)	Potential drugs			
			Methazolamide		Imatinib	
			Affinity (kcal/mol)	Rank	Affinity (kcal/mol)	Rank
Autodock Vina	Self	80	-5.5	1368/2387	-9.8	12/2387
	DLIGAND2		-7.43	1254/2387	-17.77	6/2387
Autodock4	Self	114.7	-7.05	829/2387	-9.58	22/2387
	DLIGAND2		-7.89	1212/2387	-18.1	4/2387
idock	Self	54	-5.21	1395/2387	-10.29	2/2387
	DLIGAND2		-7.1	1309/2387	-18.18	3/2387
LeDock	Self	102	-5.98	445/2387	-8.14	14/2387
	DLIGAND2		-8.57	1160/2387	-18.11	2/2387
GalaxyDock	Self	48	-12.89	1335/2387	-21.95	84/2387
	DLIGAND2		-8.66	1131/2387	-17.75	6/2387
iGEMDOCK	Self	143.4	-80.42	1370/2387	-128.26	15/2387
	DLIGAND2		-6.28	1373/2387	-16.48	7/2387

Reference

Chen P, et al. (2019) DLIGAND2: an improved knowledge-based energy function for protein－ligand interactions using the distance-scaled, finite, ideal-gas reference state, *Journal of Cheminformatics*, **11**, 1-11.

Wishart, D.S. et al. (2007) HMDB: the Human Metabolome Database, *Nucleic Acids Research*, **35**, D521-D526.

Eberhardt, et al. (2021) AutoDock Vina 1.2.0: New Docking Methods, Expanded Force Field, and Python Bindings, *Journal of Chemical Information and Modeling*, **61**, 3891-3898.

Le Guilloux, V. et al. (2009) Fpocket: an open source platform for ligand pocket detection, *BMC Bioinformatics*, **10**, 168.

Li, H. et al. (2012) idock: A multithreaded virtual screening tool for flexible ligand docking. In: 2012 IEEE Symposium on Computational Intelligence in Bioinformatics and Computational Biology (CIBCB), 2012. pp. 77-84.

Zhao, H., and Caflisch, A. (2013) Discovery of ZAP70 inhibitors by high-throughput docking into a conformation of its kinase domain generated by molecular dynamics, *Bioorganic & medicinal chemistry letters*, **23**, 5721-5726.

Mysinger, et al. (2012) Directory of Useful Decoys, Enhanced (DUD-E): Better Ligands and Decoys for Better Benchmarking, *Journal of Medicinal Chemistry*, **55**, 6582-6594.

Rego, N. and Koes, D. (2015) 3Dmol.js: molecular visualization with WebGL, *Bioinformatics*, **31**, 1322-1324.

Shin, W. and Seok, C. (2012) GalaxyDock: Protein−Ligand Docking with Flexible Protein Side-chains, *Journal of Chemical Information and Modeling*, **52**, 3225-3232.

Trott, O. and Olson, A.J. (2009) AutoDock Vina: Improving the speed and accuracy of docking with a new scoring function, efficient optimization, and multithreading, *Journal of Computational Chemistry*, **31**, 455-461.

Wishart, D. S, et al. (2018) DrugBank 5.0: a major update to the DrugBank database for 2018, *Nucleic Acids Research*, **46**, D1074-D1082.

Yang, J.M. and Chen, C.C. (2004) GEMDOCK: a generic evolutionary method for molecular docking, *Proteins*, **55**, 288-304.

Li Zilun, et al. (2022) Imatinib and methazolamide ameliorate COVID-19-induced metabolic complications via elevating ACE2 enzymatic activity and inhibiting viral entry, *Cell Metabolism*, **34**, 424-440.
